# Assessment of Cardiovascular Risk Categories and Achievement of Therapeutic Targets in European Patients with Type 2 Diabetes

**DOI:** 10.3390/jcm13082196

**Published:** 2024-04-10

**Authors:** Delia Reurean-Pintilei, Claudia-Gabriela Potcovaru, Teodor Salmen, Liliana Mititelu-Tartau, Delia Cinteză, Sandra Lazăr, Anca Pantea Stoian, Romulus Timar, Bogdan Timar

**Affiliations:** 1 Doctoral School of Medicine and Pharmacy, “Victor Babes” University of Medicine and Pharmacy, 300041 Timisoara, Romania; 2 Centre for Molecular Research in Nephrology and Vascular Disease, “Victor Babes” University of Medicine and Pharmacy, 300041 Timisoara, Romania; sandra.lazar@umft.ro (S.L.); timar.romulus@umft.ro (R.T.); bogdan.timar@umft.ro (B.T.); 3 Department of Diabetes, Nutrition and Metabolic Diseases, Consultmed Medical Centre, 700544 Iasi, Romania; 4 9th Department of Physical Medicine and Rehabilitation, “Carol Davila” University of Medicine and Pharmacy, 050474 Bucharest, Romania; 5 Doctoral School, “Carol Davila” University of Medicine and Pharmacy, 050474 Bucharest, Romania; 6 Department of Pharmacology, Faculty of Medicine, ‘Grigore T. Popa’ University of Medicine and Pharmacy, 700115 Iasi, Romania; 7 First Department of Internal Medicine, “Victor Babes” University of Medicine and Pharmacy, 300041 Timisoara, Romania; 8 Department of Hematology, Emergency Municipal Hospital Timisoara, 300041 Timisoara, Romania; 9 Diabetes, Nutrition and Metabolic Diseases Department, “Carol Davila” University of Medicine and Pharmacy, 050474 Bucharest, Romania; anca.stoian@umfcd.ro; 10 Department of Second Internal Medicine—Diabetes, Nutrition, Metabolic Diseases, and Systemic Rheumatology, “Victor Babes” University of Medicine and Pharmacy, 300041 Timisoara, Romania; 11 Department of Diabetes, Nutrition and Metabolic Diseases Clinic, “Pius Brînzeu” Emergency Clinical County University Hospital, 300723 Timisoara, Romania

**Keywords:** cardiovascular risk, therapeutic targets, dyslipidemia, hypertension targets

## Abstract

**Background:** Individuals diagnosed with type 2 diabetes mellitus (T2DM) are more prone to experiencing severe cardiovascular (CV) events, often occurring at a younger age, due to a complex interplay of risk factors. T2DM diagnosis inherently classifies patients as belonging to a higher CV risk group. In light of the increased susceptibility to severe CV outcomes, our study aims to assess the distribution of CV risk categories and the attainment of therapeutic targets among Romanian patients diagnosed with T2DM. **Methods:** A cross-sectional analysis was performed, including 885 patients diagnosed with T2DM who were consecutively admitted to a secondary care hospital unit between January and July 2019. Data collection included demographics, lipid profile, glycated hemoglobin (HbA1c), blood pressure (BP), estimated glomerular filtration rate (eGFR), and medication specifics for T2DM and associated conditions. Patients were stratified into CV risk categories based on the ESC/EAS guidelines, encompassing moderate, high, and very high risk categories. The rationale for selecting these guidelines for CV risk categories was that they were current and provided best practice recommendations for T2DM patients during the cross-sectional evaluation. We assessed therapeutic target achievement rates for LDL-C, HbA1C, and BP for each CV risk category. Additionally, we examined utilization rates of statins and novel cardio- and reno-protective, non-insulin antidiabetic medications. **Results:** The group’s average age was 62.9 ± 7.7 years and comprised 53.7% females. An average HbA1c level of 7.1 ± 1.3% was observed in the group. Within the cohort, 83% had hypertension, with a mean systolic BP of 132 ± 16.2 mm Hg and mean diastolic BP of 80 ± 9.6 mm Hg. Additionally, 64.6% of patients were obese, with a mean body mass index of 32.3 ± 5.3 kg/m^2^. Mean LDL-C levels varied across the different CV risk categories: 106.6 ± 35.6 mg/dL in the very high risk category, 113 ± 39.3 mg/dL in the high risk category, and 124.3 ± 38.3 mg/dL in the moderate risk category. Most treatment schemes included metformin (87.0%) and statins (67.0%), with variable use rates for other glucose-lowering and CV risk-modifying therapies. The percentage of patients using GLP-1 RAs was 8.1%, while 3.9% used SGLT2 inhibitors. **Conclusions:** Most Romanian patients with T2DM are at very high or high CV risk. Despite reaching glycemic control targets, most patients are not achieving the composite target, which includes, besides glycemic control, BP values and lipid profile. Many patients with T2DM are not benefiting from DM therapies with additional cardiorenal benefits or statins.

## 1. Introduction

The prevalence of type 2 diabetes mellitus (T2DM) has exceeded estimates in previous and repeated epidemiologic predictions. It has been recognized as a global public health issue with an increasing prevalence, particularly in low- and middle-income countries. T2DM complications continue to represent a significant burden on healthcare systems, while fatality rates have increased in the first two decades of the century [1].

T2DM poses a threat to cardiovascular (CV) health, necessitating effective management of this condition to lower CV risk. It is also noteworthy that the connection between T2DM and CV diseases (CVD) is bidirectional, underscoring the need for comprehensive management strategies that address both conditions. For each 1% rise in hemoglobin A1c (HbA1c), the risk of atherosclerotic CV events (CVEs) has been reported to increase by nearly one-fifth. Maintaining HbA1c levels below the recommended threshold of 7%, for over ten years, significantly reduces CVD risk, with the lowest mortality rate observed within the optimal range, just below this threshold [2]. This augmented risk is further amplified when additional standard risk factors (excess weight, hypertension, smoking, and dyslipidemia) overlap with the hyperglycemic state [3,4,5].

Ischemic coronary heart disease (ICHD) and stroke continue to be the most prevalent causes of death, according to the World Health Organization’s report on the leading ten causes of death from 2000 to 2019. In addition, T2DM is the greatest cause of increasing mortality among men and is within the ten most prevalent causes of death, with a yearly increase of 80% since 2000 [6]. Individuals with DM often experience CVEs at a younger age compared to those without the condition [7]. Although CVD incidence and mortality rates are lower in high-income countries, these nations do not represent the entire population. It is unknown to what extent these rates are changing for T2DM patients in middle- and low-income countries [8]. Compared to other Europeans, people residing in central-eastern European regions have been identified as having the most significant mortality prevalence due to CVEs, with Eastern Europeans having a shorter life expectancy, these lifespan inequalities being closely associated with variations in ICHD fatality rates. The fact that Eastern European countries with high prevalences of ICHD have increased rates of coronary revascularization is not sufficient to equally reduce the mortality caused by ischemic coronary pathologies in all these countries [9,10,11,12,13,14,15,16]. As the causal link between CVEs and exposure to high levels of low-density lipoprotein-cholesterol (LDL-C) has been established [17,18], recent studies have evaluated the degree to which LDL-C targets were being met in different European populations, taking into consideration the CV risk category of the patients included. In Poland and Romania, only slightly over a third of these patients met the 2019 European Society of Cardiology/European Atherosclerosis Society’s (ESC/EAS) recommended LDL-C target. Compared to other countries, a more significant proportion of patients in Romania had a DM diagnosis [16].

In Romania, DM is estimated to affect 11.6% of persons between the ages of 20 and 79, and an additional 2.4% of the population has undiagnosed DM, according to the findings of the national PREDATORR study conducted in 2016 [19]. A recent analysis assessed the rates and mortality causes related to DM in a large cohort of Romanian adults aged between 20 and 64 years. CVD was confirmed to be the leading cause of death in this group, with a greater impact among younger age groups [20]. The World Heart Federation reported that, in 2019, Romania experienced 150,427 fatalities resulting from CVDs [21].

The continuous effort for optimal CV risk stratification in patients with DM from 2019 to 2023 exemplifies the commitment to individualizing risk categories and delivering personalized care. The 2019 ESC/EAS guidelines identified T2DM patients as having increased CV risk, deeming additional risk estimation systems, including SCORE, unnecessary, as the T2DM diagnosis itself signifies heightened risk. Therefore, in 2019, patients with DM were identified as having very high risk, high risk or moderate risk [3]. The 2021 ESC Guidelines on CVD prevention recommended using the ADVANCE or DIAL models for CVD risk assessment in patients with DM [4]. However, these models may not be completely feasible in all varying risk profiles across different demographics in Europe. The 2023 ESC guidelines update the CV risk assessment for patients with DM and advise on using the SCORE2-Diabetes algorithm for determining 10-year CVD risk in T2DM individuals over 40 years old, without CVD or target organ damage (TOD). The SCORE2-Diabetes model is a tool that considers various factors to determine CVD risk in patients with DM, using metrics such as age, gender, smoking habits, blood pressure (BP), cholesterol, and renal health to stratify risk into low/moderate/high/very high categories [5].

As reducing the burden of CVD in people with T2DM remains a goal that is yet to be achieved, current guidelines consider this and add to T2DM management, besides blood sugar-lowering recommendations, a multifactorial personalized approach that also targets CV risk factors. Guidelines emphasize the management of well-known CV risk factors, including BP, lipid profile, and weight management, while simultaneously preserving optimal glycemic control. These specific therapeutic objectives that target CVD prevention must be continuously integrated into managing T2DM patients to avoid clinical inertia. Following the best clinical practice recommendations, taking measures such as prompt screening, comprehensive medical examination, and assessment of comorbidities are clearly outlined in the guidelines. To develop effective preventative measures in this population, it is necessary to initially evaluate each patient’s CV risk profile. The next step is to assess whether the patient achieves the multifactorial therapeutic targets according to the CV risk category in which he/she is included and to select the class of novel T2DM medication with CV benefits that may bring additional positive outcomes [22,23,24,25].

This research aimed to assess the distribution of CV risk categories based on the ESC/EAS guidelines in a cohort of Romanian patients with T2DM. Additionally, the study aimed to evaluate to what extent patients achieved the therapeutic target for LDL-C, HbA1C, and BP levels. At the same time, it evaluated to what extent novel DM therapies with cardiorenal protection and statins were used in this cohort.

## 2. Materials and Methods

### 2.1. Study Design and Patients

This was a single-center, cross-sectional, consecutive-case, population-based study conducted following the Declaration of Helsinki and approved by the Institutional Ethics Committee of Consultmed Hospital in Iași County, Romania (protocol number CMD102018006, from 18 October 2018), a healthcare facility providing services included in the national health insurance program. Between January and July 2019, the first 1000 patients meeting the inclusion criteria, presented in Table 1, were consecutively invited to participate in this study during their routine scheduled clinic visit at Consultmed Hospital.

**Figure 1:**
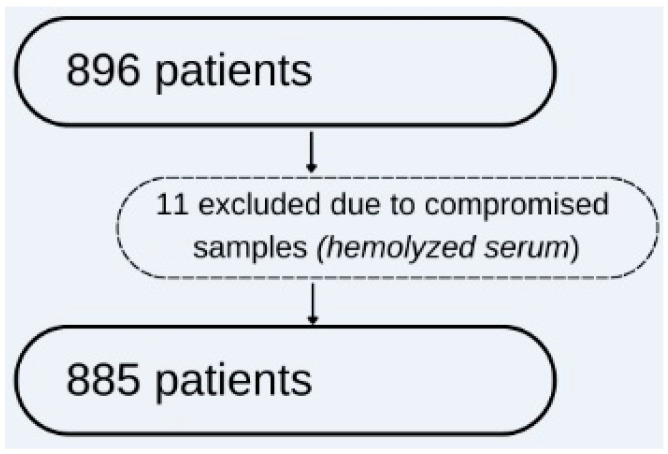
Study flow-chart reporting the number of patients.

Medical records were collected over a single enrolment visit to obtain data, and no formal study visits were subsequently performed. Out of a total of 896 patients who provided their informed consent, 11 patients were excluded due to extreme laboratory results with a high chance of compromised blood samples or laboratory analyzer errors, as seen in Figure 1.

We analyzed data from the remaining 885 patients, which was deemed sufficient. Our sample size was determined based on the Iasi County population, reported as 944,074 citizens in its Statistical Annuary [26], with a margin of error of 3.29% and a 95% confidence level, ensuring a representative sample size of at least 885 patients.

Patient data were compiled from hospital discharge reports and included DM duration, demographics (age, gender, residential background), anthropometrics (height, weight, body mass index (BMI)), concurrent conditions (e.g., hypertension, dyslipidemia, and atherosclerotic CVD (ASCVD)), laboratory test results (HbA1c, LDL-C, total cholesterol (total-C), high-density lipoprotein-cholesterol (HDL-C), triglycerides (TG), uric acid, and estimated glomerular filtration rate (eGFR)), and treatment specifics (such as antidiabetics, antihypertensive agents, antiplatelet, and lipid-lowering medications). T2DM diagnosis and medication were validated through referral notes from the general practitioner, which the patient presented at the time of evaluation, and was further corroborated by the patient’s medical history. The inclusion criteria for this study specifically required that participants had been diagnosed with the condition for at least 3 months before being incorporated into the study. Information on concurrent conditions was obtained from the same referral documents provided by the general practitioner and medical history, encompassing diagnoses such as hypertension along with prescribed medications, dyslipidemia with respective treatments, and ASCVD. This category included a history of myocardial infarction, stable angina, revascularization procedures, cerebrovascular events, peripheral arterial disease (PAD), and atherosclerosis exceeding 50% stenosis as identified through imaging techniques. Laboratory tests: blood samples were drawn from the cubital vein between 7:00 and 9:00 AM following an overnight fasting, as per local protocols. Height was measured using a stadiometer, with heels against the wall, back straight, and eyes forward for accurate posture. Weight was recorded using a calibrated scale, ensuring patients were minimally clothed and without shoes for precision. BMI was calculated by dividing weight in kilograms by the square of height in meters. Obesity was defined as a BMI exceeding 30 kg/m^2^.

### 2.2. Patient Stratification

Patients were assigned to CV risk categories based on their CV risk profile, as assessed by the ESC/EAS guidelines applicable at that moment (the 2019 ESC/EAS classification) into moderate, high, and very high risk groups. The analysis did not include the low risk category since, according to the aforementioned guidelines, having T2DM shifts patients to the moderate risk category. Consequently, as per the 2019 ESC/EAS guidelines, utilizing the SCORE system for classification was not advised.

T2DM patients were identified as having a very high CV risk if they had a previous diagnosis of CVD, or any of the following: TOD, including microalbuminuria, eGFR < 30 mL/min/1.73 m^2^, neuropathy, retinopathy, or a minimum of three traditional risk factors (smoking, obesity, hypertension, dyslipidemia). A previous CVD diagnosis was defined by documented ASCVD: history of acute coronary syndrome, stable angina, coronary or other arterial territories revascularization procedures, stroke, transient ischemic attack, or PAD, or significant atherosclerosis (stenosis over 50% in two main epicardial arteries on coronary angiography or computed tomography), or via carotid ultrasound.

Patients included in the high risk category had DM for more than 10 years or had one additional risk factor, without TOD.

The moderate risk category encompassed patients with T2DM under the age of 50, diagnosed with T2DM for less than a decade, and lacking other risk factors.

The therapeutic objectives for each CV risk category were established based on the 2019 ESC/EAS standards for LDL-C, and the 2019 American Diabetes Association (ADA) guidelines for hemoglobin A1c (HbA1C) and BP:

i. Moderate risk category: LDL-C < 100 mg/dL, HbA1c < 7%, BP < 130/80 mmHg.
ii. High risk category: LDL-C < 70 mg/dL, HbA1c < 7%, and BP < 130/80 mmHg.
iii. Very high risk category: LDL-C < 55 mg/dL, HbA1c < 7%, and BP < 130/80 mmHg.

Furthermore, we evaluated the use of statin alongside novel DM therapies from therapeutic classes that demonstrated additional cardiorenal benefits (GLP-1 RAs and SGLT2i), which were available in Romania in 2019.

### 2.3. Statistical Analysis

Patient data were systematically recorded in an Excel database, utilizing its functions for statistical analysis. This involved calculating averages, means, medians, and standard deviations to determine central tendencies, and counts to quantify specific occurrences within the dataset. These methods provided a detailed understanding of the patient data.

## 3. Results

The 885-patient cohort’s demographics included a mean age of 62.9 ± 7.7 years, and 53.7% were females. The mean duration of T2DM was 9.0 ± 4.4 years. The detailed patient characteristics are presented in Table 2. The prevalence of obesity in the study population was 64.6%, the included patients having a mean BMI of 32.3 ± 5.3 kg/m^2^; 83% of patients had hypertension, with a mean systolic BP (SBP) of 132 ± 16.2 mm Hg and mean diastolic BP (DBP) of 80 ± 9.6 mm Hg. Data regarding smoking were not available in the electronic database. The mean HbA1c level was 7.1% ± 1.3, while the lipid profile included a mean total-C of 185.1 ± 43.3 mg/dL, a mean HDL-C of 44.9 ± 11.8 mg/dL, a mean LDL-C of 107.73 ± 36.01 mg/dL, and a median TG level of 142 mg/dL (104 to 197 interquartile range). ASCVD was present in 13.9% of patients, and the LDL-C mean values’ distribution was 106.6 ± 35.6 mg/dL in the very high CV risk category, 113.0 ± 39.3 in the high CV risk category and, respectively, 124.3 ± 38.3 mg/dL in the moderate CV risk category.

In addition, Table 2 provides a summary of the prescribed glucose-lowering therapies by category: metformin (87.0%), insulin (25.2%), dipeptidyl peptidase four inhibitors (13.0%), GLP-1 RAs (8.1%), and SGLT2i (3.9%). Furthermore, the table details the use of other therapies targeting CV risk factors: statins (67.0%) and angiotensin-converting enzyme inhibitors/angiotensin receptor blockers (ACEI/ARBs) (61.5%).

We identified 821 patients in the very high CV risk category, 10 in the high risk category, and 54 in the moderate risk category. This information is presented in Table 3, Table 4 and Table 5. Additionally, these tables include data regarding the number of patients achieving treatment targets for each category, namely LDL-C, HbA1C, and BP, both individually and in combination with the innovative antidiabetic and lipid-lowering medication utilized.

## 4. Discussion

This study specifically examined the distribution of various CV risk categories among contemporary patients with T2DM in an Eastern European context. It also evaluated the rates of achieving LDL-C targets based on the 2019 ESC/EAS guidelines and the targets for HbA1C and BP established by the 2019 ADA guidelines. Furthermore, the study assessed the rates at which novel non-insulin antidiabetic medications, such as GLP-1 RAs and SGLT2i, as well as statins, are being used across these risk categories.

Our cohort exhibits unmodifiable risk variables such as age, gender, and duration of DM, while the modifiable risk factors include BP, obesity, and dyslipidemia. The group’s median age is relatively lower than expected, averaging 62.9 ± 7.7 years. Patients’ glycemic control is relatively well-maintained, with HbA1c levels averaging 7.1% ± 1.3% after 9 years of exposure to the disease (9.0 ± 4.4 years). Vintila et al. recently described a similar cohort of T2DM patients, yet of an older age of 71 years, and a satisfactory mean HbA1c value of 7.2% [27].

This study’s demographic reflects broader trends, with a significant proportion of the patients, nearly 65%, living with obesity, with an average BMI of 32.3 ± 5.3 kg/m^2^. Obesity rates have been reported to be higher in Eastern Europe, in particular, with rates reaching or exceeding 20% [28]. Obesity is a significant risk factor that can be influenced to decrease the likelihood of developing DM. Approximately 90% of persons diagnosed with T2DM fall into the overweight or obesity categories. Living with excess weight corresponds to a threefold increase in the development of DM, while obesity is associated with a 7-fold increase [29,30,31].

As expected, this excess weight coexists with hypertension at a high prevalence of 83% of participants. For Romania, the prevalence of hypertension among patients with T2DM is reported to be greater than the prevalence estimated for the overall population [32]. In a recent paper evaluating a population with similar demographics, an association between adiposity and arterial hypertension was found to be positive. At the same time, a direct correlation was identified between high BP and T2DM [33].

When compared to reports on standard of care evaluations in populations from the same time frame in Scotland and Denmark, our patients were younger (62 years compared to 67 years and 72 years, respectively), with a longer DM duration (9.0 years versus 7.8 years), with a lower proportion having a prior CVE (13.9% compared to 32% and 21.4%, respectively), slightly lower HbA1c values, similar SBP and BMI, and lower percentages of patients on a statin (67% versus 75% and 78%) and on ACEi/ARBs (61% versus 83% and 75%, respectively) [34,35].

The lipid profile assessment provides a detailed overview of the extent to which targets are being met in this cohort. The average total-C level is 185.1 ± 43.3 mg/dL, HDL-C at 44.9 ± 11.8, and LDL-C at 107.7 ± 36.0 mg/dL. Taking a closer look at LDL-C values across each CV risk group, disparities are revealed, with an LDL-C level of 106.6 ± 35.6 mg/dL in the very high CV risk category, 113.0 ± 39.3 mg/dL in the high CV risk category, and 124.3 ± 38.3 mg/dL in moderate CV risk category.

The Santorini study, encompassing data from 9044 patients from 14 European countries, none of them from Eastern Europe, reported a median LDL-C level of 82 mg/dL in the overall population, 93 mg/dL in the high-risk patient group, and 78 mg/dL in the very-high-risk patient group as evaluated by physicians. Ray et al. [36] found that 73.3% of patients were not at their 2019 ESC/EAS risk-based LDL-C goal. Further, upon re-evaluation at the central level, the CV risk for patients was categorized as follows: 6.5% were classified as high risk and 91.0% were designated as very high risk, according to the 2019 ESC/EAS guidelines [36]. In our population analysis, almost 93% were discovered to fall within the very high risk category based on the same classification system.

ASCVD affected 13.9% of the patients included in our study. McGurnaghan et al. [34] described a proportion of over 30% of the population studied as having had a previous CVE. In individuals with very high CV risk, only 0.7% achieved the LDL-C, BP, and HbA1c targets simultaneously, despite 50.4% reaching the HbA1c target or 27.5% meeting the BP target. Additionally, in individuals who attained all three objectives, namely, LDL-c, BP, and HbA1c, the novel antidiabetic drugs were prescribed only once each for SGLT2i and GLP-1 RAs.

In our study, 5.0% of those classified within the very high CV risk category met their LDL-C targets as outlined in the 2019 guidelines, mirroring findings from the DA VINCI study, which found a 4% achievement rate among primary prevention patients in the same risk category [16]. This contrasts with the 11% attainment rate under the 2016 ESC/EAS guidelines, a discrepancy attributed to evolving guideline criteria. Romanian participants constituted 12% of the DA VINCI study’s 2154 patient cohort [16]. The DA VINCI findings also highlight a greater likelihood of achieving LDL-C targets among very-high-risk patients under secondary prevention [16], suggesting a potential underestimation of risk by physicians for patients without prior CVEs, possibly leading to hesitancy in escalating lipid-lowering treatments. In our study, it was observed that 67.8% of patients from the high risk category received statin as a lipid-lowering therapy. This is higher than the 48.4% reported in the Santorini study [34]. The findings of the Morieri et al. [37] study indicated that 15% of the patients in the very high risk category were on statin therapy and achieved their LDL-C target, as opposed to 2.7% in our study. According to recent research, reducing LDL-C levels by 40 mg with a statin drug can result in a 9% decrease in overall mortality rates and a 21% reduction in major CVEs [38]. Since 2017, the American Association of Clinical Endocrinologists (AACE) has recommended an LDL-cholesterol target below 55 mg/dL for individuals in the “extreme risk category” [39]. In 2019, European professional societies harmonized their guidelines, indicating that individuals categorized as “very high risk” for primary or secondary prevention should strive for a target of less than 55 mg/dL [3]. Still, several research groups consistently report a low success rate in attaining these objectives, demonstrating the critical need for increased awareness and more aggressive management strategies to improve lipid target achievement rates in high-risk populations [16,27,36,37].

In our cohort, a small number of patients in the high (0.2%) and moderate (0.6%) CV risk categories were able to simultaneously achieve their LDL-C, BP, and HbA1c goals. Statin and innovative antidiabetic medications were rarely prescribed in these categories.

In Romania in 2019, according to the national prescription protocols for medical service providers contracted by the national insurance system, the local prescribing conditions required the initiation of innovative antidiabetic medication classes to be conditional on an HbA1c value over 7%. There were also several other limitations related to combinations with other antidiabetics and the timing of the specific combinations [40]. Our cohort’s low utilization rate of GLP1 RAs and SGLT2 inhibitors can be attributed mainly to the unique regional factors mentioned above. Overall, in our entire cohort, the utilization of SGLT2i was at 3.9%, DPP-4i at 13%, and GLP-1 RAs at 8.1%. There was a marked tendency for prescribing these medications in patients belonging to the very high risk category. In 2020, Vencio et al. [41] aimed to examine the current usage of DM medicines with evidence for CV benefit in patients with T2DM. The study reported that slightly over 20% of patients were prescribed either an SGLT2i or a GLP-1 RA, with 15% of patients on SGLT2i and 9% on GLP-1 RA [41]. Arnold et al. [42] conducted the Discover observational study, analyzing data from 38 nations. The researchers noted that the prescription rates for GLP1 RAs and SGLT2-i were suboptimal. Although there was a rise in utilization from 10.8% at enrolment to 16.1% at the end of the follow-up time frame, there were notable differences among the various countries. A utilization rate exceeding 63% was observed in comparison to countries with weaker economic resources [42]. Metformin and insulin prescriptions were found to be lower in the Scottish and Danish cohort analyses (57% and 54% versus 87% in our patients, respectively) and (11% and 19.5%, versus 25%, respectively), while SGLT2i use was comparable to the Danish study, 4%, or lower, 2%, in Scottish patients. GLP1 RAs were used by 8% of our patients, compared to 5.4% of the patients from Denmark and 2.7% of the patients from Scotland. In the Scottish cohort, sulphonylurea usage was 26%, in the Danish cohort it was 8%, and within our patient data, it was 13% [34,35].

The findings of our study are very important in determining the number of people with DM who do not have access to therapies for preventing CVD. Our analysis shows that, in Romania, people with DM still face challenges in meeting their treatment objectives across numerous areas, as of 2019. This study highlights the need for improved management strategies to address all aspects of the multifactorial approach required for treating T2DM. In a recent survey of 10 Eastern and Southern European countries on implementing ADA/EASD recommendations, the authors concluded that although physicians reported high adherence to guidelines, local protocols, medication access, and reimbursement restrictions would need to be addressed [43].

The data presented underline that there is still potential for targeted interventions to improve patient care quality. This aspect is broadly compatible with prior regional research, but more data on the subject still needs to be collected. Recent updates in medical research have led to a revised perception and understanding that patients are continuously exposed to a silently evolving and dynamic disease, specifically DM, characterized especially by the heightened risk of CVEs and mortality. In particular, restratifying CV risk in patients with T2DM is crucial for identifying those who are most in need of timely interventions. Employing various imaging techniques, such as coronary computed tomography angiography, magnetic resonance imaging, and others, can enhance the identification of CVD risk, and this approach is applicable in both primary and secondary prevention strategies. The utilization of these advanced imaging methods allows for a more detailed evaluation of atherosclerotic disease, myocardial damage, and overall CV health, which can play a pivotal role in the management and prevention strategies for high-risk patients [44]. As atherosclerosis is a persistent process that affects all regions of the vasculature, it is essential to improve the assessment of parameters related to the early and accelerated progression of plaque formation, instability, and rupture. Lipoprotein(a) (Lp(a)) serves as an exacerbating factor across the entire spectrum of CV risk, from primary to secondary prevention, to established CVE. It also represents a treatment target for the newest lipid-lowering agents including proprotein convertase subtilisin/kexin type 9 inhibitors (PCSK9i), small interfering RNA (siRNA), and inhibitory antisense oligonucleotides (ASOs) [45]. However, the CV benefits of these medications are still under investigation. The ESC/EAS 2019 guidelines recommend measuring Lp(a) at least once in adults to detect high-risk patients and refine CV risk stratification. However, challenges in implementing these guidelines include physicians’ limited awareness of the recommendations and the associated costs. Future research may concentrate on the cost-effectiveness of improving CV risk assessment by including this analysis [3].

Our study provides an important glimpse of modern, real-world DM management, compared to the multifaceted strategy advised by the guidelines. However, further work is needed to improve and ensure that therapeutic goals are met.

An identified limitation of our study is the incompleteness of data pertaining to certain risk variables, such as smoking, which the patients reported with insufficient detail. An additional constraint is the absence of data regarding the explanations for why individuals with heightened risk factors appear to receive insufficient therapy, specifically whether this is due to substandard prescription practices, non-compliance, or adverse-effect complications. The large sample’s representation of the Romanian population is significant to our research and the longitudinal observation of this cohort is crucial for assessing the adherence to existing clinical guidelines and for evaluating the progress towards therapeutic goal attainment in these patients’ clinical courses. This strategy marks a novel trajectory for our research, grounded in the present data.

## 5. Conclusions

Most patients with T2DM living in Romania are at very high or high CV risk. Despite advances in therapeutic options, the guideline’s recommendations are challenging to obtain in a real-world scenario, especially regarding lipid targets. In contrast, glycemic and BP targets are more frequently achieved. Considering that the majority of patients with DM are at very high and high CV risk, to improve their overall prognosis, particular emphasis should be given to the evaluation of treatment success using composite metrics containing at least all the three components evaluated in this study: glycemic control, BP, as well as lipid profile. Despite guideline recommendations, there is an unmet need regarding the use of DM therapies with additional cardiorenal benefits, especially in patients with T2DM at high or very high CV risk.

## Figures and Tables

**Table 1:** Inclusion and exclusion criteria.

Inclusion Criteria	Exclusion Criteria
Adults >18 years of age	<18 years of age
Informed consent provided	Pregnant or breastfeeding females
Positive diagnosis of T2DM for ≥3 months	Positive diagnostic different than T2DM (such as Type 1 DM, secondary diabetes, or latent autoimmune DM)
Standard of care for the associated conditions for ≥3 months	Positive test for autoimmune markers (IA2A, ICA, GADA, ZnT8 AB antibodies)
	Declined to sign the informed consent form

DM—diabetes mellitus; T2DM—type 2 diabetes mellitus.

**Table 2:** Patient characteristics.

Characteristic	*n* = 885
Demographics	
Age (years), mean (SD)	62.9 ± 7.7
Women, %, (*n*)	53.7% (475)
DM mean duration, mean (SD)	9.0 ± 4.4
Risk factors	
BMI (kg/m2), mean (SD)	32.3 ± 5.3
Obesity, %, (*n*)	64.6% (572)
SBP (mm Hg), mean (SD)	132 ± 16.2
DBP (mm Hg), mean (SD)	80 ± 9.6
HBP, %, (*n*)	83% (737)
HbA1c (%), mean (SD)	7.1 ± 1.3
Total-C (mg/dL), mean (SD)	185.1 ± 43.3
HDL-C (mg/dL), mean (SD)	44.9 ± 11.8
TG (mg/dL), median (interquartile range)	142 (104 to 197)
LDL-C (mg/dL), mean (SD)	107.7 ± 36.0
Atherosclerotic CVD, %, (*n*)	13.9% (123)
eGFR (mL/min/1.73 m^2^)	87.5 ± 20.6
LDL-C in very high CV risk category, (mg/dL), mean (SD)	106.6 ± 35.6
LDL-C in high CV risk category, (mg/dL), mean (SD)	113 ± 39.3
LDL-C in moderate CV risk category, (mg/dL), mean (SD)	124.3 ± 38.3
Glucose-lowering medications usage	
Insulin, %, (*n*)	25.2% (223)
Metformin, %, (*n*)	87.0% (687)
DPP-4i, %, (*n*)	13.0% (115)
GLP-1 RAs, %, (*n*)	8.1% (71)
SGLT2i, %, (*n*)	3.9% (34)
SU, %, (*n*)	13.1% (116)
Other therapies	
ACEi/ARBs, %, (*n*)	61.5% (544)
Statin, %, (*n*)	67.0% (593)

SD—standard deviation; BMI—body mass index; SBP—systolic blood pressure; DBP—diastolic blood pressure; HBP—high blood pressure; total-C—total cholesterol; HDL-C—high-density lipoprotein cholesterol; TG—triglycerides; LDL-C—low-density lipoprotein cholesterol; CVD—cardiovascular disease; CV—cardiovascular; DPP-4i—dipeptidyl peptidase four inhibitors; GLP-1 RAs—glucagon-like peptide one receptor agonist; SGLT2i—sodium-glucose cotransporter-2 inhibitors; ACEI/ARBs—angiotensin-converting enzyme inhibitors/angiotensin receptor blockers.

**Table 3:** Very high risk CV category, attainment of treatment targets according to 2019 ESC/EAS Guidelines for LDL-C, 2019 ADA Guidelines for HbA1C and BP, and prescription rates of innovative antidiabetic and statin medications.

Treatment Target for Patients with Very High CV Risk Category (*n* = 821)	Patients Achieving Target	Patients with SGLT2i Prescription	Patients with GLP-1 RAs Prescription	Patients with Statin Prescription
LDL-C < 55 mg/dL, %, (*n*)	5.0% (41)	0.3% (3)	0.8% (7)	2.7% (22)
HbA1c < 7%, %, (*n*)	50.4% (446)	1.4% (12)	2.7% (24)	35.0% (310)
BP < 130/80 mmHg, %, (*n*)	27.5% (243)	0.8% (7)	2.4% (21)	20.3% (180)
LDL-C < 55 mg/dL + HbA1c < 7%, %, (*n*)	2.3% (20)	0.2% (2)	0.5% (4)	1.2% (11)
HbA1c < 7% + BP < 130/80 mmHg, %, (*n*)	15.5% (137)	0.5% (4)	0.6% (5)	11.3% (100)
LDL-C < 55 mg/dL+ BP < 130/80 mmHg, %, (*n*)	1.5% (13)	0.1% (1)	0.3% (3)	0.6% (5)
LDL-C < 55 mg/dL + HbA1c < 7% + BP < 130/80 mmHg, %, (*n*)	0.7% (6)	0.1% (1)	0.1% (1)	0.2% (2)

CV—cardiovascular; LDL-C—low density lipoprotein cholesterol; BP—blood pressure; SGLT2i—sodium-glucose cotransporter-2 inhibitors; GLP-1 RAs—glucagon-like peptide 1 receptor agonist.

**Table 4:** High CV risk category, attainment of treatment targets according to 2019 ESC/EAS Guidelines for LDL-C, 2019 ADA Guidelines for HbA1C and BP, and prescription rates of innovative antidiabetic and statin medications.

Treatment Target for Patients with High CV Risk Category (*n* = 10)	Patients Achieving Target	Patients with SGLT2i Prescription	Patients with GLP-1 RAs Prescription	Patients with Statin Prescription
LDL-C < 70 mg/dL, %, (*n*)	0.2% (2)	0	0	0
HbA1c < 7%, %, (*n*)	0.9% (8)	0	0	0.5% (4)
BP < 130/80 mmHg, %, (*n*)	0.7% (6)	0	0	0.5% (4)
LDL-C < 70 mg/dL + HbA1c< 7%, %, (*n*)	0.2% (2)	0	0	0
HbA1c < 7% + BP < 130/80 mmHg, %, (*n*)	0.6% (5)	0	0	0.3% (3)
LDL-C < 70 mg/dL+ BP <130/80 mmHg, %, (*n*)	0.2% (2)	0	0	0
LDL-C < 70 mg/dL + HbA1c < 7% + BP < 130/80 mmHg, %, (*n*)	0.2% (2)	0	0	0

CV—cardiovascular; LDL-C—low density lipoprotein cholesterol; BP—blood pressure; SGLT2i—sodium-glucose cotransporter-2 inhibitors; GLP-1 RAs—glucagon-like peptide 1 receptor agonist.

**Table 5:** Moderate CV risk category, attainment of treatment targets according to 2019 ESC/EAS Guidelines for LDL-C, 2019 ADA Guidelines for HbA1C and BP, and prescription rates of innovative antidiabetic and statin medications.

Treatment Target for Patients with Moderate CV Risk Category (*n* = 54)	Patients Achieving Target	Patients with SGLT2i Prescription	Patients with GLP-1 RAs Prescription	Patients with Statin Prescription
LDL-C < 100 mg/dL, %, (*n*)	1.6% (14)	0.1% (1)	0% (0)	0.7% (6)
HbA1c < 7%, %, (*n*)	3.6% (32)	0	0.1% (1)	1.9% (17)
BP < 130/80 mmHg, %, (*n*)	2.0% (18)	0	0.2% (2)	1.0% (9)
LDL-C < 100 mg/dL + HbA1c < 7%, %, (*n*)	0.8% (7)	0	0	0.3% (3)
HbA1c < 7% + BP < 130/80 mmHg, %, (*n*)	1.2% (11)	0)	0.1% (1)	0.6% (5)
LDL-C < 100 mg/dL+ BP < 130/80 mmHg, %, (*n*)	0.8% (7)	0	0	0.3% (3)
LDL-C < 100 mg/dL + HbA1c < 7% + BP < 130/80 mmHg, %, (*n*)	0.6% (5)	0	0	0.2% (2)

CV—cardiovascular; LDL-C—low density lipoprotein cholesterol; BP—blood pressure; SGLT2i—sodium-glucose cotransporter-2 inhibitors; GLP-1 RAs—glucagon-like peptide 1 receptor agonist.

## Data Availability

Data are contained within the article.
